# Supplementary material for: Coordination modulation of hydrated zinc ions to enhance redox reversibility of zinc batteries
Source: Nat Commun. 2023 Jun 14;14:3526. doi: 10.1038/s41467-023-39237-3 (PMC10267194; doi:10.1038/s41467-023-39237-3)
Supplement: Supplementary file 1 — Supplementary Information [file 41467_2023_39237_MOESM1_ESM.pdf]

Supplementary Information

Coordination modulation of hydrated zinc ions to enhance redox reversibility of zinc batteries

Song Chen[1], Deluo Ji[1], Qianwu Chen[1], Jizhen Ma[1], Shaoqi Hou[2] and Jintao Zhang[1,*]

[1]Key Laboratory for Colloid and Interface Chemistry, Ministry of Education, School of Chemistry and Chemical Engineering, Shandong University, Jinan 250100, China. E-mail: jtzhang@sdu.edu.cn

[2]School of Mathematical and Physical Sciences, University of Technology Sydney, Ultimo, New South Wales 2007

List of Contents

1. Supplementary Figures:

Supplementary Fig. 1 a) Voltage-time curves of Zn-Zn symmetric cells in $ZnSO_4$ electrolytes with various concentrations at 1 mA cm^{-2}, 1 mAh cm^{-2} and b) the enlarged detail.

Supplementary Fig. 2 a) Voltage-time curves of Zn-Zn symmetric cells in $Zn(PS)_2$ electrolytes with various concentrations at 1 mA cm^{-2}, 1 mAh cm^{-2} and b) the enlarged detail.

Supplementary Fig. 3 Voltage-time curves of Zn-Zn symmetric cells in various electrolytes at 3 mA cm^{-2}, 3 mAh cm^{-2}.

Supplementary Fig. 4 Nucleation and platform overpotentials at the Zn anode.

Supplementary Fig. 5 Rate performance of Zn symmetric cells at the current density of 0.5, 1, 2, 5 and 10 mA cm^{-2} with the charge-discharge capacity of 0.25, 0.5, 1, 2.5 and 5 mAh cm^{-2}.

Supplementary Fig. 6 SEM images of Zn foil after 10 cycles in different electrolytes at 1 mA cm^{-2}, 1 mAh cm^{-2}. a, b) 1 M $ZnSO_4$, c, d) 1 M $Zn(PS)_2$, e, f) 1 M $Zn(PS)_2$+0.2 TBATS.

Supplementary Fig. 7 Optical images of Zn deposited at a current density of 5 mA cm^{-2} in a) 1 M $ZnSO_4$, b) 1 M $Zn(PS)_2$ and c) 1 M $Zn(PS)_2$+0.2 TBATS electrolytes, respectively.

Supplementary Fig. 8 SEM images of a, c, e) Zn surface and the b, d, f) cross-section image after zinc deposition of 5 mAh cm^{-2} in different electrolytes: a, b) 1 M $ZnSO_4$, c, d) 1 M $Zn(PS)_2$, e, f) 1 M $Zn(PS)_2$+0.2 TBATS.

Supplementary Fig. 9 In situ optical images of the Zn deposition process at a current density of 5 mA cm^{-2} in 1 M $Zn(PS)_2$ electrolyte.

Supplementary Fig. 10 Chronoamperometry of Zn anode in different electrolytes under -80 mV within 400 s.

Supplementary Fig. 11 Coulombic efficiency (CE) test of Cu-Zn asymmetric cells in a) 1 M $ZnSO_4$, b) 1 M $Zn(PS)_2$ and c) 1 M $Zn(PS)_2$+0.2 TBATS, respectively.

Supplementary Fig. 12 Optical image of glass vial for the corrosion test.

Supplementary Fig. 13 SEM images of Zn foil after being immersed in a) 1 M $ZnSO_4$ b) 1 M $Zn(PS)_2$ and c) 1 M $Zn(PS)_2$+0.2 TBATS for one week.

Supplementary Fig. 14 XRD pattern of Zn foils after immersion test.

Supplementary Fig. 15 FTIR spectra of Zn foils immersed in solutions for one week.

Supplementary Fig. 16 SEM images of Zn foils in a) 1 M $ZnSO_4$ b) 1 M $Zn(PS)_2$ and c) 1 M $Zn(PS)_2$+0.2 TBATS solutions and corresponding d-f) EDX.

Supplementary Fig. 17 a) Tafel plots in different electrolytes and b) the corresponding corrosion current.

Supplementary Fig. 18 Enlarged CV curves of Ti-Zn asymmetric cells in different electrolytes.

Supplementary Fig. 19 The solvation-shell in diluted and concentrated $Zn(PS)_2$ electrolytes.

Supplementary Fig. 20 Raman spectra of $ZnSO_4$ with different concentrations.

Supplementary Fig. 21 The configurations and binding energies of a) Zn^{2+}-H_2O, b) Zn^{2+}-SO_4^{2-}, c) SO_4^{2-}-H_2O, d) Zn^{2+}-PS^-, and e) PS^--H_2O complexes conducted by DFT calculation.

Supplementary Fig. 22 a) The MD snapshot of 1 M $ZnSO_4$ and the electrolyte structure. b) The RDF g(r) and coordination number N(r) of Zn-O_w and Zn-O_{anion}.

Supplementary Fig. 23 The size of a) SO_4^{2-} and b) PS^- anions.

Supplementary Fig. 24 LSV curves of Ti-Zn asymmetric cells with different electrolytes.

Supplementary Fig. 25 Nyquist plots of Ti-Ti cells in $Zn(PS)_2$ electrolyte a, c) with/without additives. b, d) The calculated ions conductivity from Nyquist plots.

Supplementary Fig. 26 Electrochemical impedance spectroscopy (EIS) results for Zn-Zn symmetric cells at a) pristine state and b) after 10 th cycle.

Supplementary Fig. 27 Electrochemical impedance spectroscopy (EIS) results with changing temperatures in a) 1 M $ZnSO_4$ and b) 1 M $Zn(PS)_2$ electrolytes.

Supplementary Fig. 28 Current-time plots and electrochemical impedance spectroscopy for the measurement of Zn^{2+} transference number in different electrolytes. a) 1 M $ZnSO_4$, b) 1 M $Zn(PS)_2$ and c) 1 M $Zn(PS)_2$+0.2 TBATS electrolytes after polarization at a constant potential (25 mV) for 3000 s. The insets are the equivalent circuit diagram and the impedance spectra before and after the polarization.

Supplementary Fig. 29 a, b) SEM images, c) XRD pattern and d) Raman spectra of PANI nanofibers.

Supplementary Fig. 30 Linear fitting between the peak current and the square root of the scan rates of the CV curves for PANI-Zn batteries.

Supplementary Fig. 31 CV curves and corresponding plots of log (peak current) vs log (scan rate) of PANI-Zn batteries with a, b) 1 M $ZnSO_4$ c, d) 1 M $Zn(PS)_2$ and e, f) 1 M $Zn(PS)_2$+0.2 TBATS electrolytes.

Supplementary Fig. 32 Electrochemical impedance spectra of PANI-Zn batteries at the pristine state.

Supplementary Fig. 33 SEM images of Zn anode of PANI-Zn batteries after 1000 cycles with a) 1 M $ZnSO_4$ b) 1 M $Zn(PS)_2$ and c) 1 M $Zn(PS)_2$+0.2 TBATS electrolytes.

Supplementary Fig. 34 In-situ Raman spectra of PANI cathode in a) 1 M $ZnSO_4$ and b) 1 M $Zn(PS)_2$ electrolytes.

Supplementary Fig. 35 a) The full XPS spectra and core-level spectra of b) Cl 2*p*, c) Zn 2*p*, d) S 2*p*, e) N 1*s* of PANI at various status in 1 M $Zn(PS)_2$+0.2 TBATS electrolyte. f) the corresponding changes of N components.

Supplementary Fig. 36 a) Comparison of CV curves of PANI-Zn batteries at 1 mV s^{-1} with 1 M $Zn(PS)_2$+0.2 TBATS electrolyte in H_2O and DMF. b) Nyquist plot of PANI-Zn batteries and c) the enlarged curves in the high-frequency range. d) Rate performance.

Supplementary Fig. 37 The proposed redox reaction of PANI in a) $Zn(PS)_2$ and b) $ZnSO_4$ electrolytes.

Supplementary Fig. 38 SEM images of PANI cathode at a) initial state and photographs of seperator after 1000 cycles in b) 1 M $ZnSO_4$, c) 1 M $Zn(PS)_2$ and d) 1 M $Zn(PS)_2$+0.2 TBATS electrolytes.

Supplementary Fig. 39 XRD patterns and Raman spectra of PANI cathode after 1000 cycles in different electrolytes.

2. *Supplementary Tables:*

Supplementary Table 1 The voltage hysteresis comparison of Zn-Zn symmetric cell with 1 M $Zn(PS)_2$+0.2 TBATS electrolyte at 1 mA cm^{-2}, 1 mAh cm^{-2} with recently reports.

Supplementary Table 2 Element contents of Zn foil surface immersed in solutions for one week.

Supplementary Table 3 Fitting results of Raman spectra of $ZnSO_4$ electrolyte.

Supplementary Table 4 Fitting results of Raman spectra of $Zn(PS)_2$ electrolyte.

Supplementary Table 5 The fitted R_{ct} of Zn symmetric cells in 1 M $ZnSO_4$ electrolyte.

Supplementary Table 6 The fitted R_{ct} of Zn symmetric cells in 1 M $Zn(PS)_2$ electrolyte.

Supplementary Table 7 The fitted R_{ct} of Zn symmetric cells in 1 M $Zn(PS)_2$+0.2 TBATS electrolyte.

Supplementary Table 8 The electrochemical performance comparison of PANI-Zn batteries based on different electrolytes.

Supplementary Table 9 Assignment of Raman peaks of the PANI-Zn batteries with different electrolytes.

1. Supplementary Figures

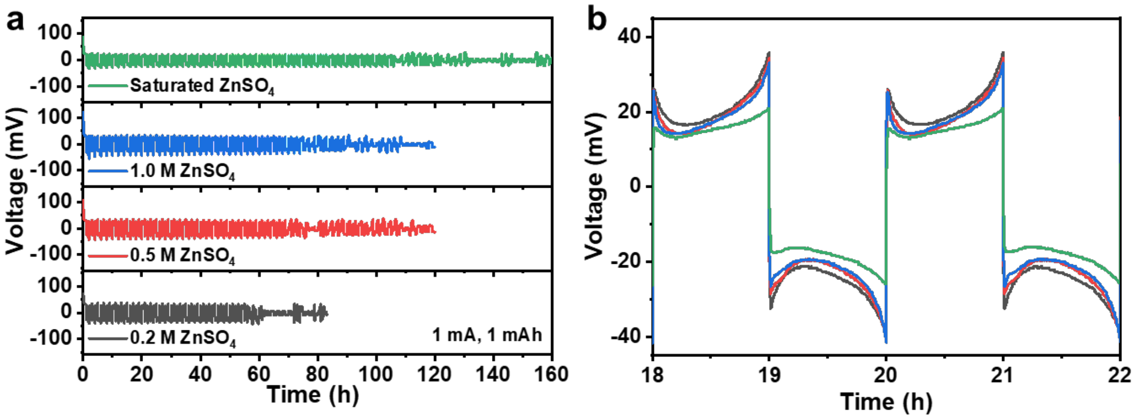

Supplementary Fig. 1 a) Voltage-time curves of Zn-Zn symmetric cells in $ZnSO_4$ electrolytes with various concentrations at 1 mA cm^{-2}, 1 mAh cm^{-2} and b) the enlarged detail.

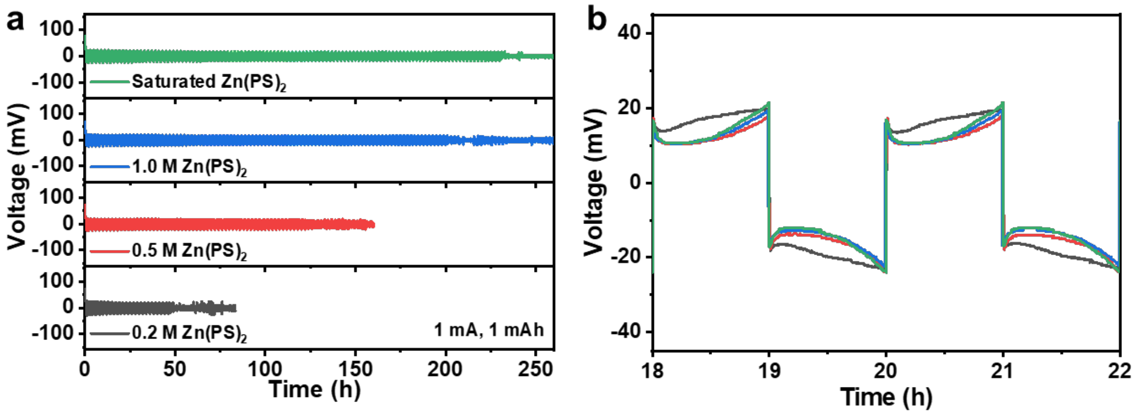

Supplementary Fig. 2 a) Voltage-time curves of Zn-Zn symmetric cells in $Zn(PS)_2$ electrolytes with various concentrations at 1 mA cm^{-2}, 1 mAh cm^{-2} and b) the enlarged detail.

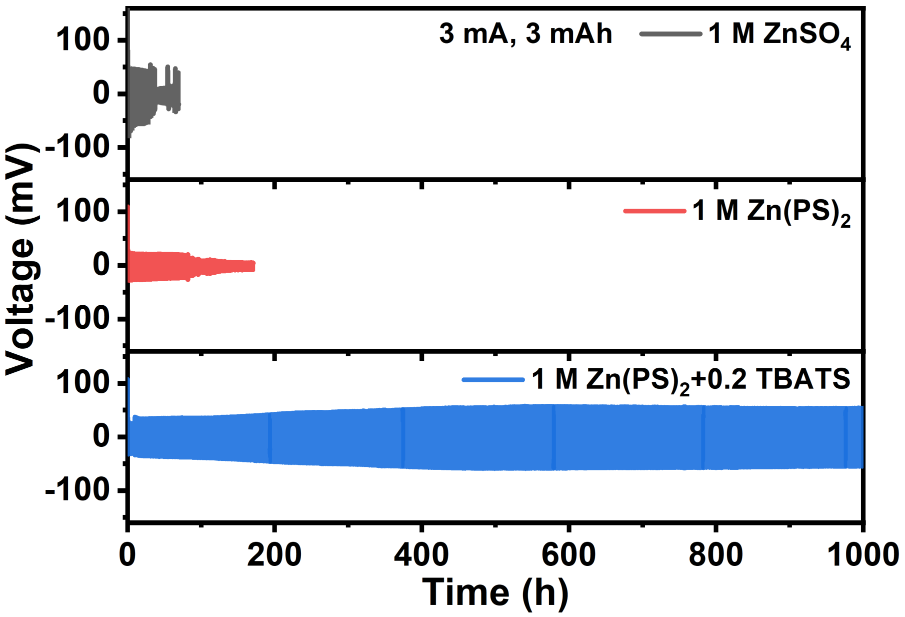

Supplementary Fig. 3 Voltage-time curves of Zn-Zn symmetric cells in various electrolytes at 3 mA cm^{-2}, 3 mAh cm^{-2}.

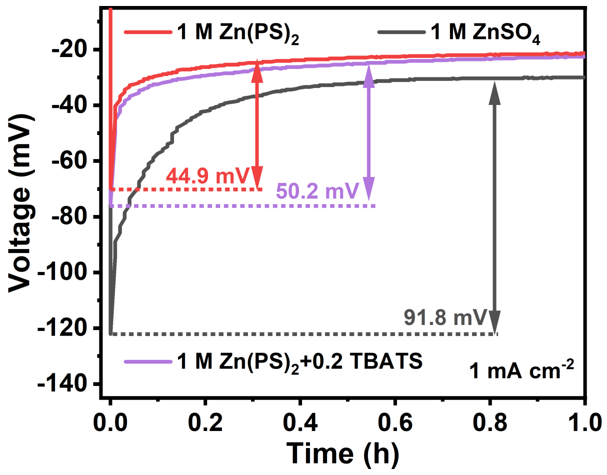

Supplementary Fig. 4 Nucleation and platform overpotentials at the Zn anode.

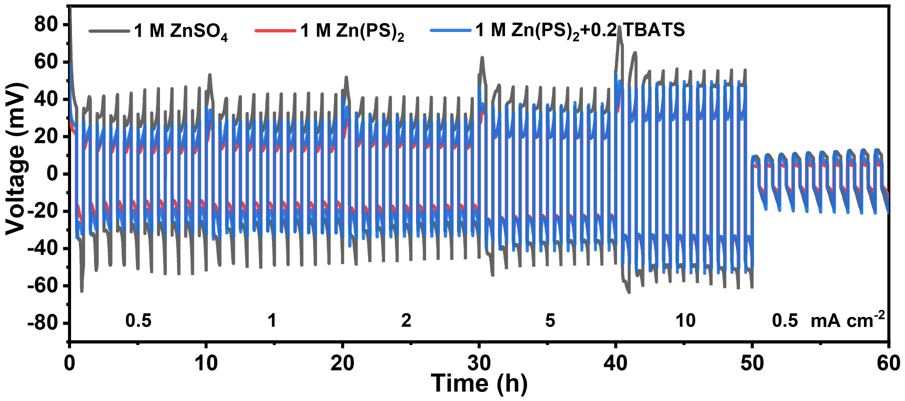

Supplementary Fig. 5 Rate performance of Zn symmetric cells at the current density of 0.5, 1, 2, 5 and 10 mA cm^{-2} with the charge-discharge capacity of 0.25, 0.5, 1, 2.5 and 5 mAh cm^{-2}.

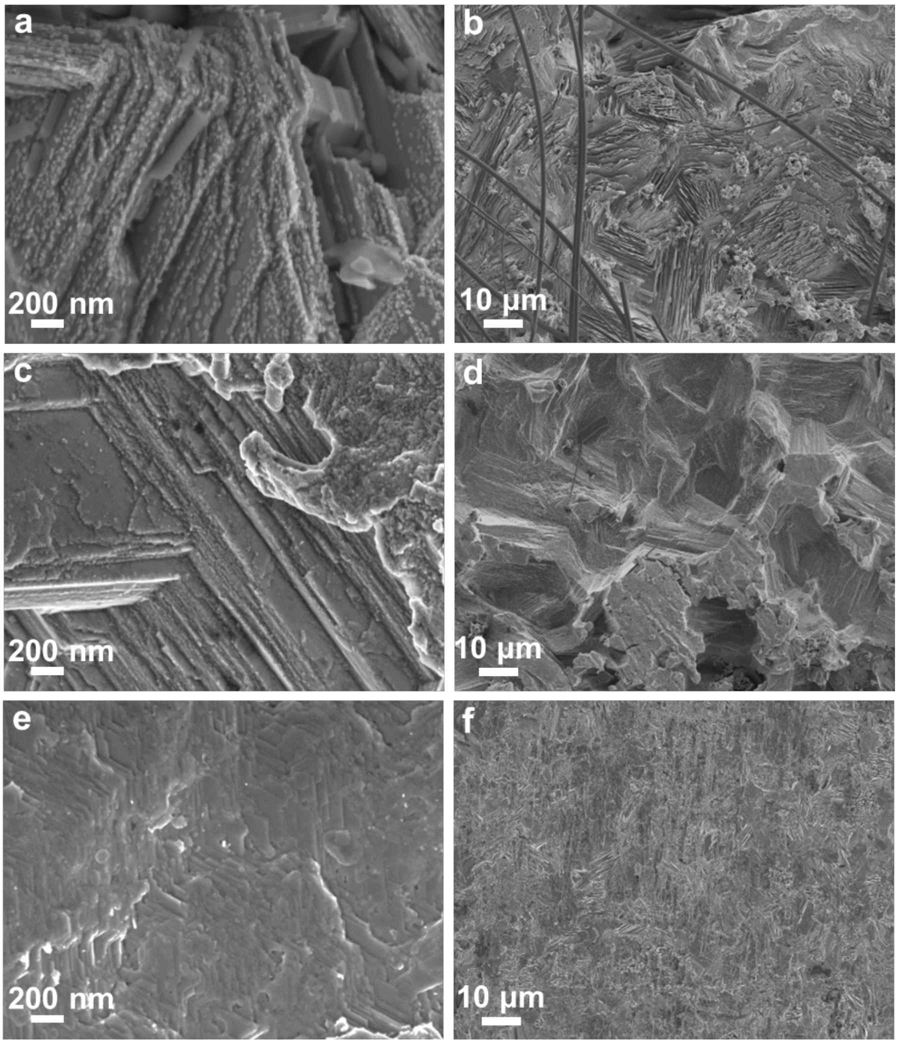

Supplementary Fig. 6 SEM images of Zn foil after 10 cycles in different electrolytes at 1 mA cm^{-2}, 1 mAh cm^{-2}. a, b) 1 M $ZnSO_4$, c, d) 1 M $Zn(PS)_2$, e, f) 1 M $Zn(PS)_2$+0.2 TBATS.

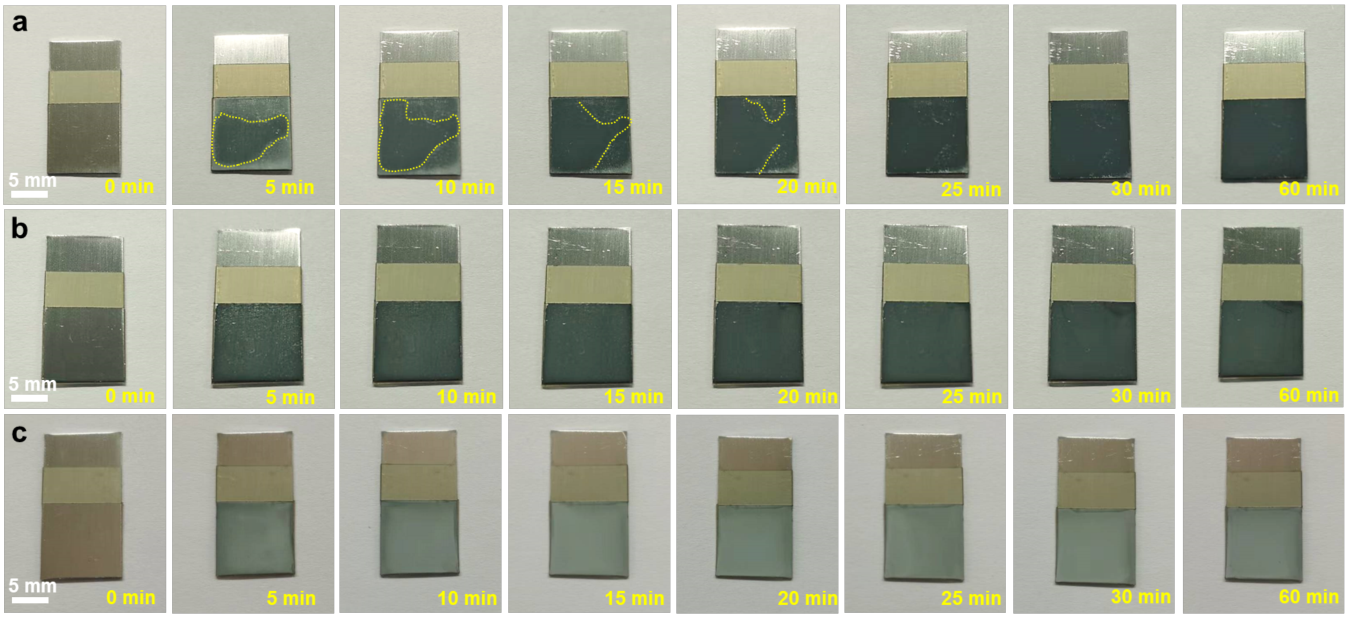

Supplementary Fig. 7 Optical images of Zn deposited at a current density of 5 mA cm^{-2} in a) 1 M $ZnSO_4$, b) 1 M $Zn(PS)_2$ and c) 1 M $Zn(PS)_2$+0.2 TBATS electrolytes, respectively.

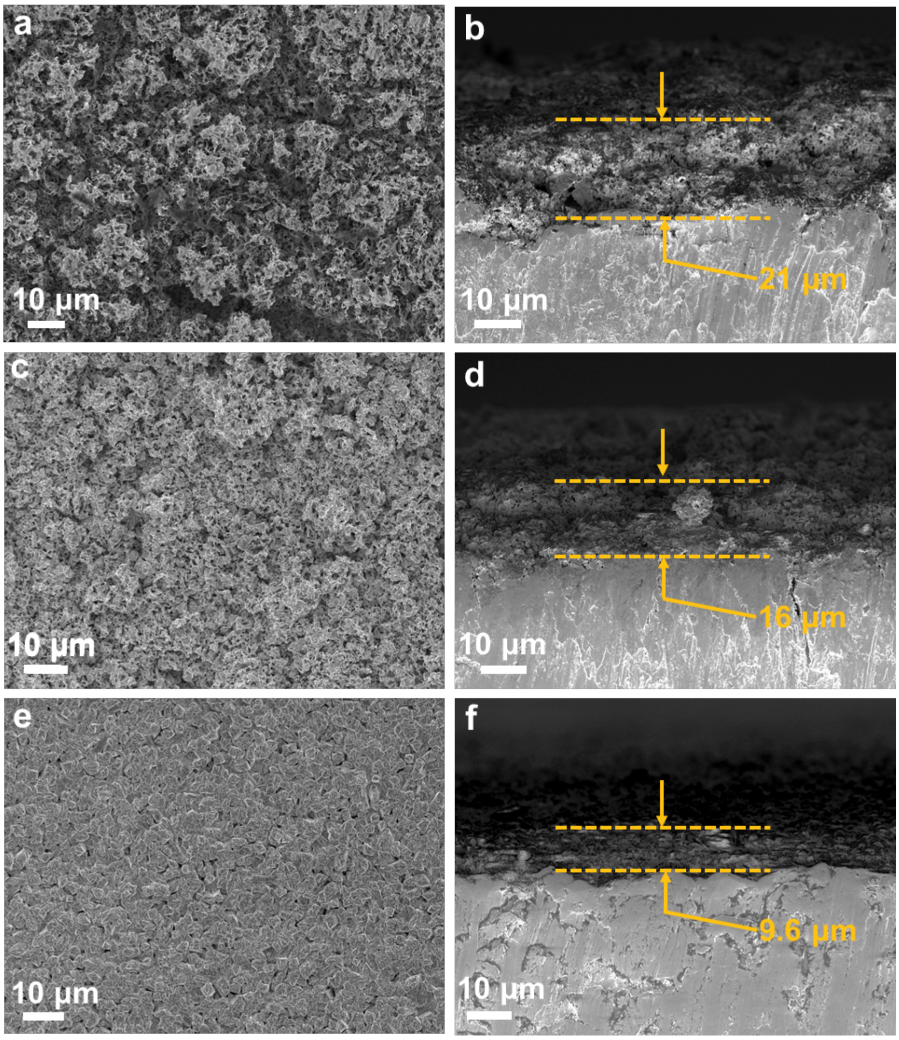

Supplementary Fig. 8 SEM images of a, c, e) Zn surface and the b, d, f) cross-section image after zinc deposition of 5 mAh cm^{-2} in different electrolytes: a, b) 1 M $ZnSO_4$, c, d) 1 M $Zn(PS)_2$, e, f) 1 M $Zn(PS)_2$+0.2 TBATS.

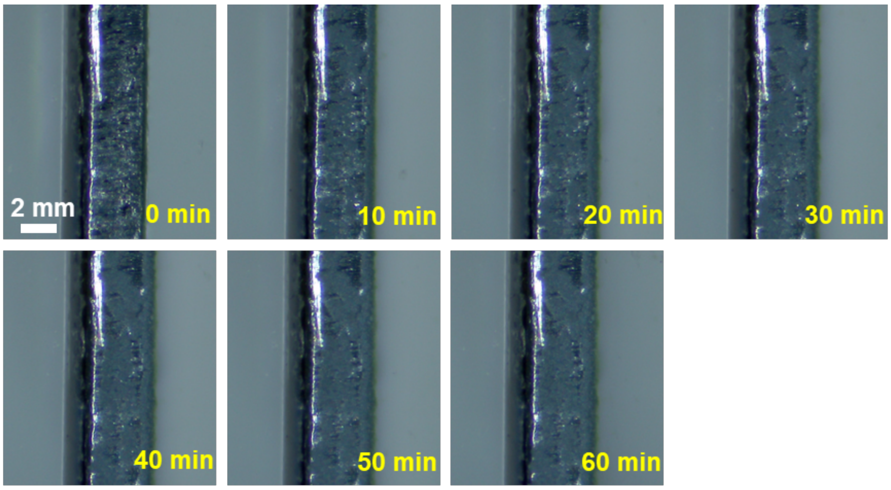

Supplementary Fig. 9 In situ optical images of the Zn deposition process at a current density of 5 mA cm^{-2} in 1 M $Zn(PS)_2$ electrolyte.

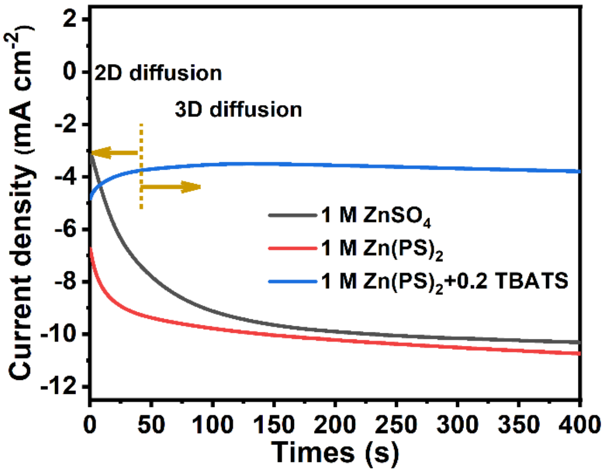

Supplementary Fig. 10 Chronoamperometry of Zn anode in different electrolytes under -80 mV within 400 s.

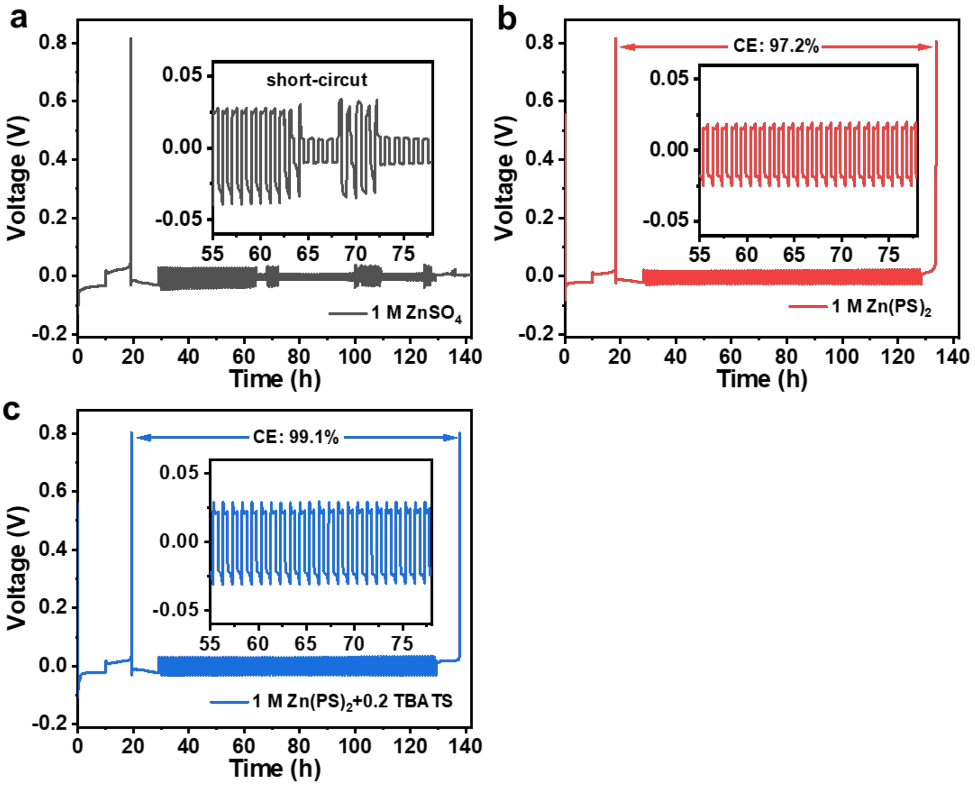

Supplementary Fig. 11 Coulombic efficiency (CE) test of Cu-Zn asymmetric cells in a) 1 M $ZnSO_4$, b) 1 M $Zn(PS)_2$ and c) 1 M $Zn(PS)_2$+0.2 TBATS, respectively.

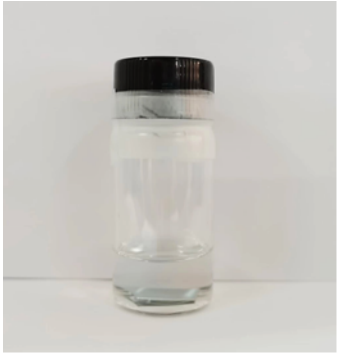

Supplementary Fig. 12 Optical image of glass vial for the corrosion test.

In order to eliminate the influence of dissolved oxygen in the electrolyte, nitrogen is bubbled continuously into the electrolyte for 30 min before the corrosion test. Then, zinc foil was put into the electrolyte and sealed in the glass vial for one week.

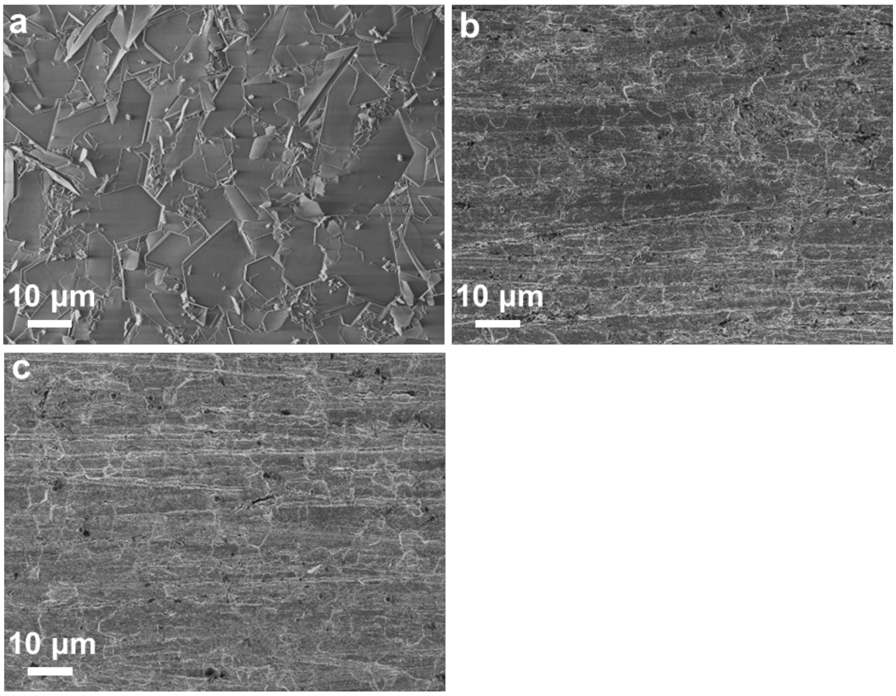

Supplementary Fig. 13 SEM images of Zn foil after being immersed in a) 1 M $ZnSO_4$ b) 1 M $Zn(PS)_2$ and c) 1 M $Zn(PS)_2$+0.2 TBATS for one week.

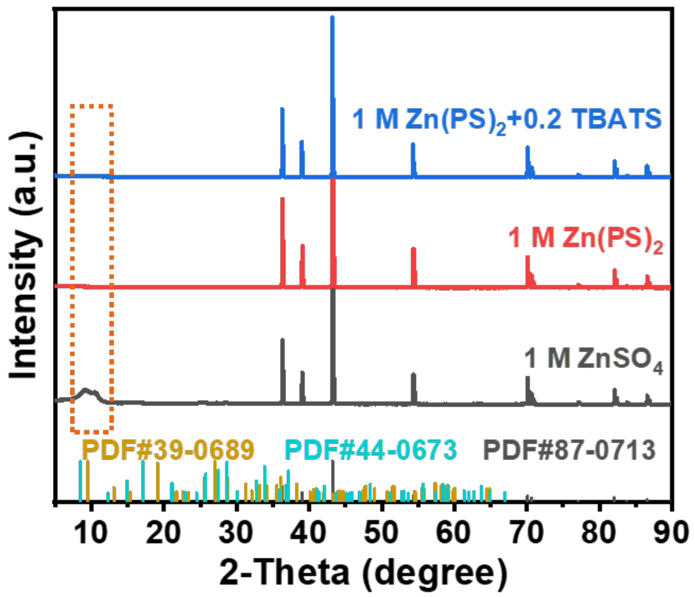

Supplementary Fig. 14 XRD pattern of Zn foils after immersion test.

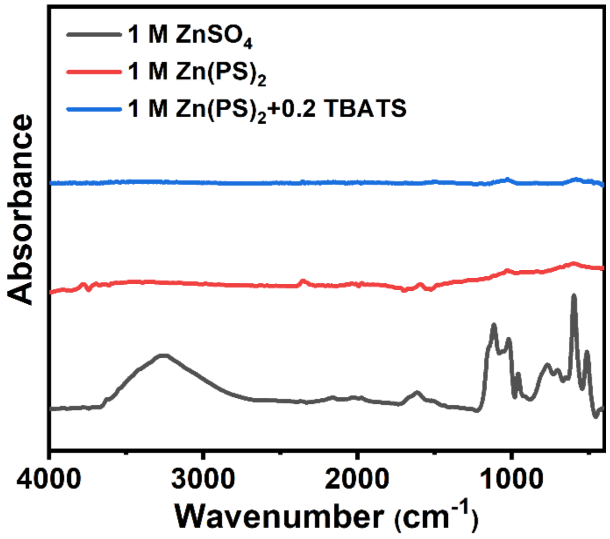

Supplementary Fig. 15 FTIR spectra of Zn foils immersed in solutions for one week.

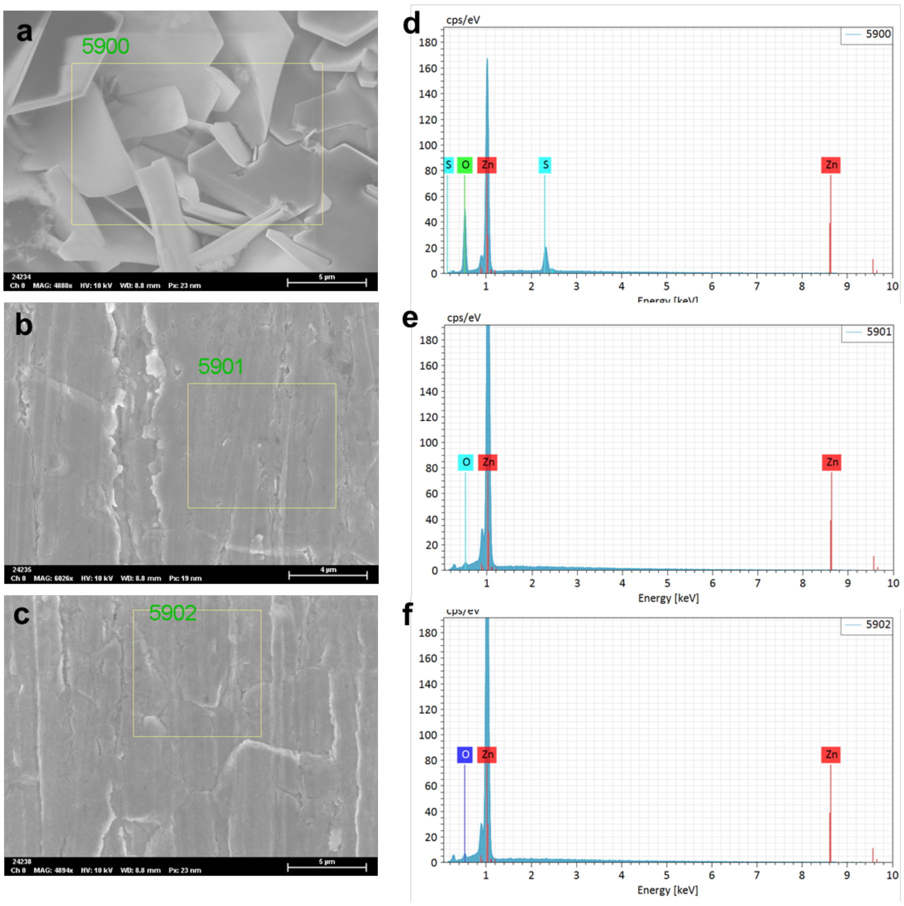

Supplementary Fig. 16 SEM images of Zn foils in a) 1 M $ZnSO_4$ b) 1 M $Zn(PS)_2$ and c) 1 M $Zn(PS)_2$+0.2 TBATS solutions and corresponding d-f) EDX.

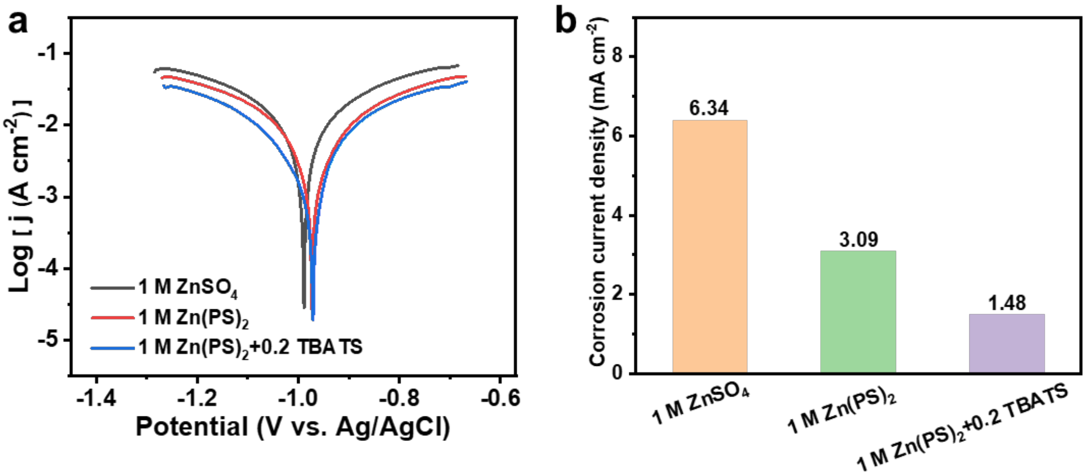

Supplementary Fig. 17 a) Tafel plots in different electrolytes and b) the corresponding corrosion current.

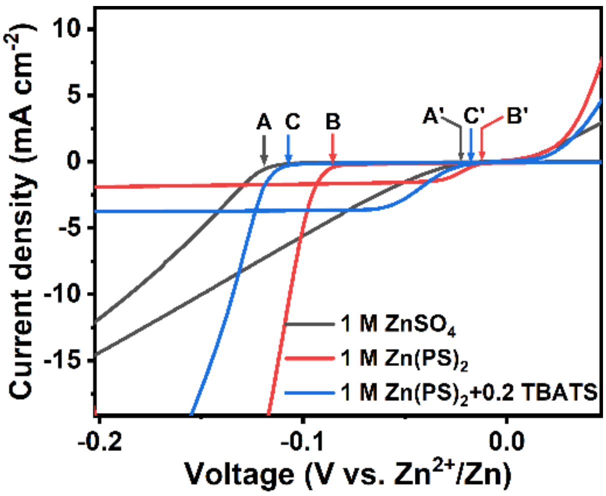

Supplementary Fig. 18 Enlarged CV curves of Ti-Zn asymmetric cells in different electrolytes.

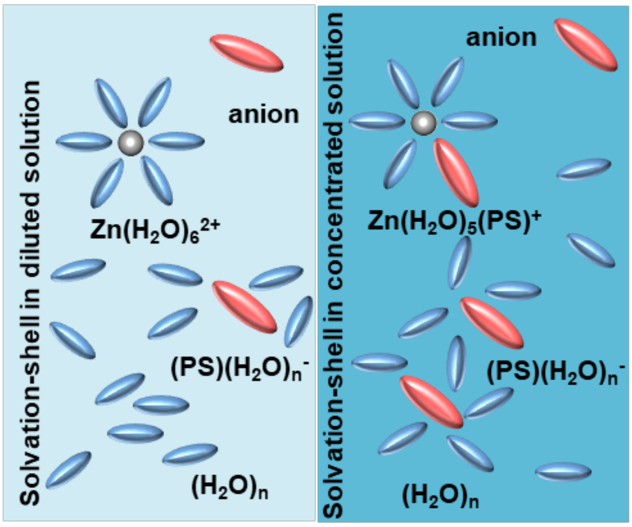

Supplementary Fig. 19 The solvation-shell in diluted and concentrated $Zn(PS)_2$ electrolytes.

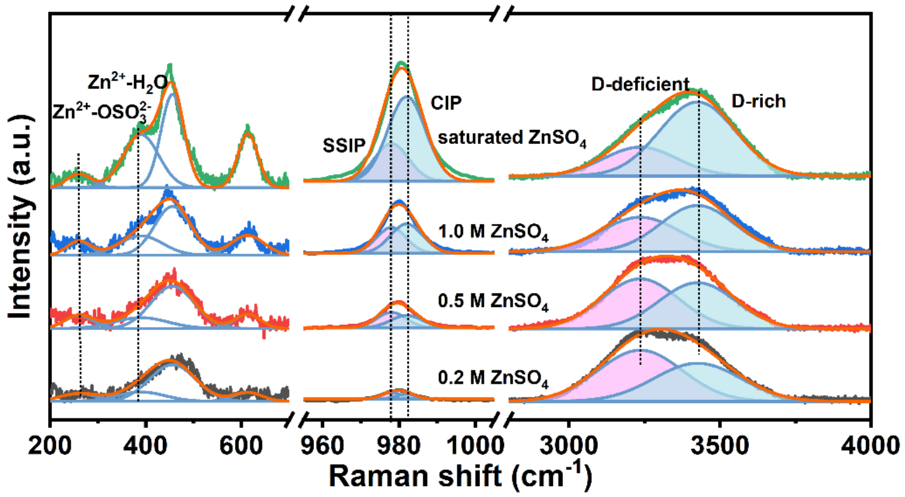

Supplementary Fig. 20 Raman spectra of $ZnSO_4$ with different concentrations.

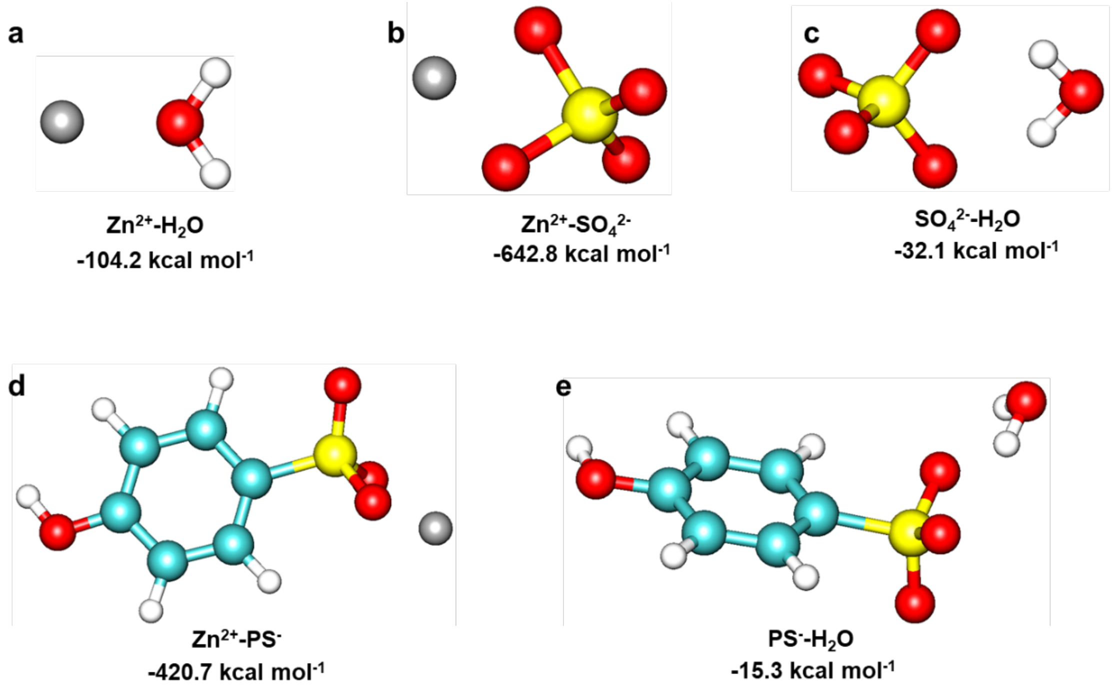

Supplementary Fig. 21 The configurations and binding energies of a) Zn^{2+}-H_2O, b) Zn^{2+}-SO_4^{2-}, c) SO_4^{2-}-H_2O, d) Zn^{2+}-PS^-, and e) PS^--H_2O complexes conducted by DFT calculation.

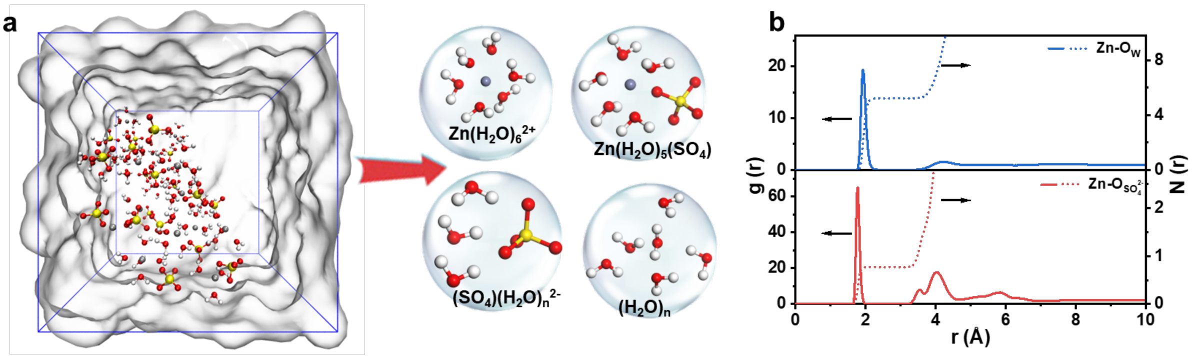

Supplementary Fig. 22 a) The MD snapshot of 1 M $ZnSO_4$ and the electrolyte structure. b) The RDF g(r) and coordination number N(r) of Zn-O_w and Zn-O_{anion}.

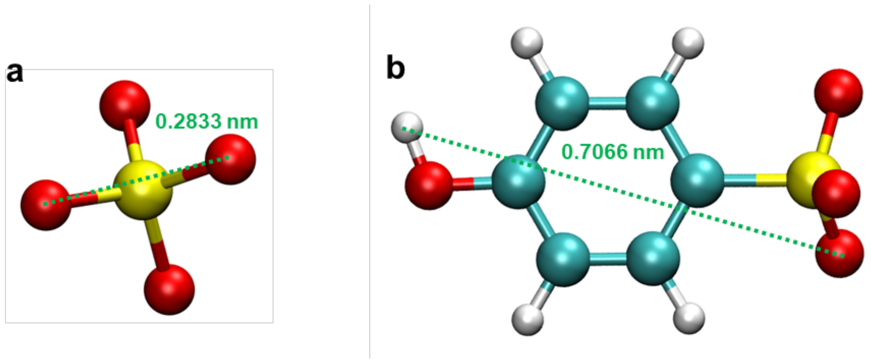

Supplementary Fig. 23 The size of a) SO_4^{2-} and b) PS^- anions.

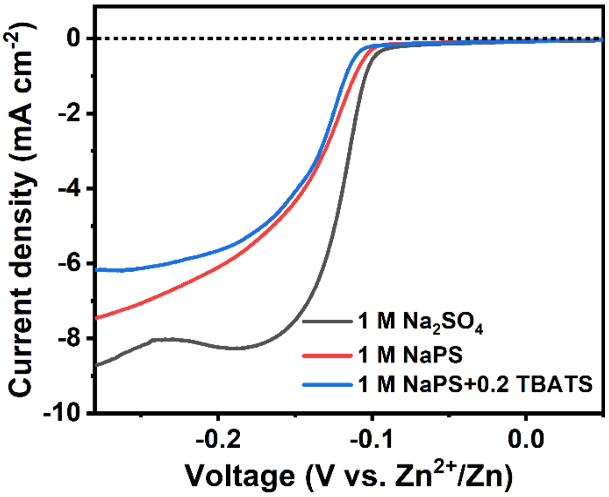

Supplementary Fig. 24 LSV curves of Ti-Zn asymmetric cells with different electrolytes.

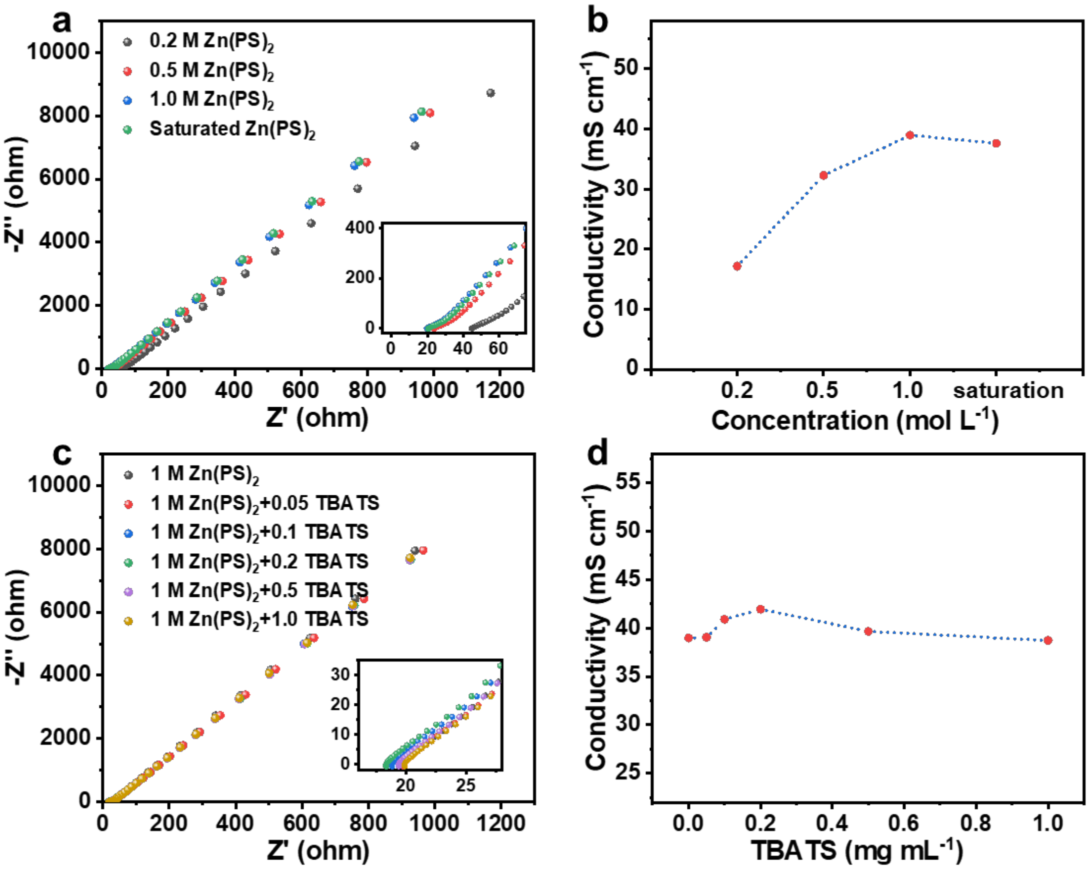

Supplementary Fig. 25 Nyquist plots of Ti-Ti cells in $Zn(PS)_2$ electrolyte a, c) with/without additives. b, d) The calculated ions conductivity from Nyquist plots.

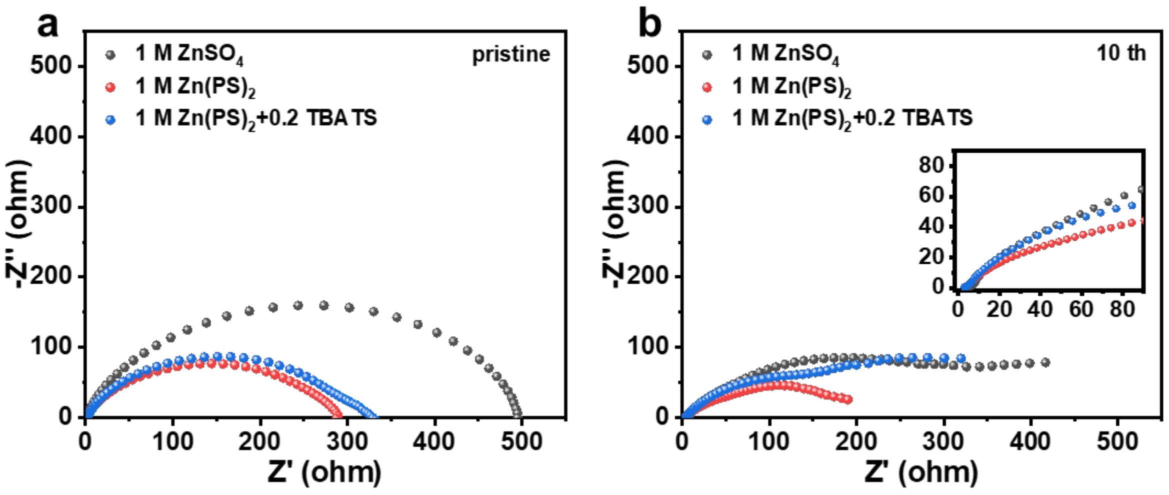

Supplementary Fig. 26 Electrochemical impedance spectroscopy (EIS) results for Zn-Zn symmetric cells at a) pristine state and b) after 10 th cycle.

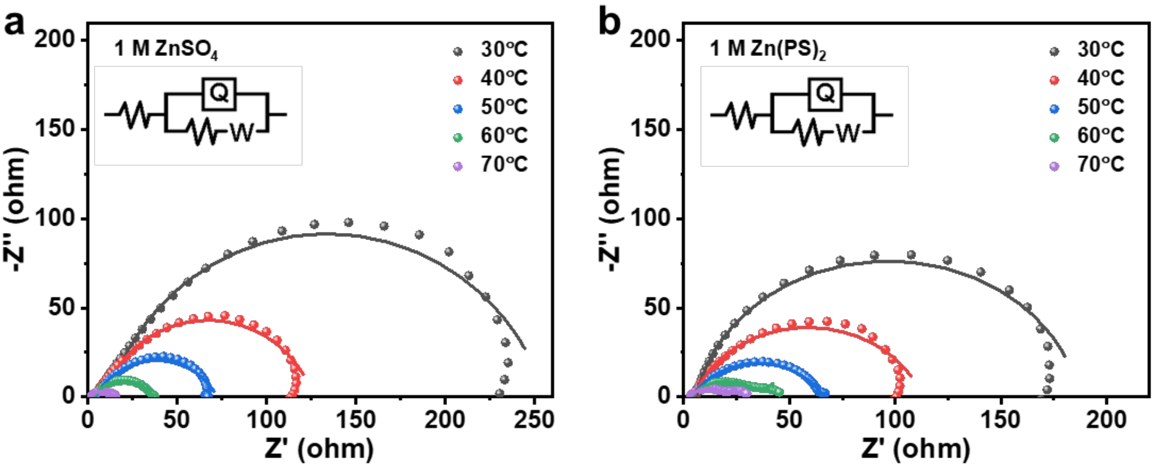

Supplementary Fig. 27 Electrochemical impedance spectroscopy (EIS) results with changing temperatures in a) 1 M $ZnSO_4$ and b) 1 M $Zn(PS)_2$ electrolytes.

The EIS results were performed in three-electrode system with Zn foil as work electrode, Ag/AgCl as reference electrode, Pt plate as counter electrode in 1 M $ZnSO_4$, 1 M $Zn(PS)_2$ electrolytes with/ without TBATS, respectively. In the temperature range of 30 to 70°C with a step of 10°C, the plots of ln (R_{ct}^{-1}) vs. $1/T$ were obtained from the EIS results according to the Arrhenius equation:

$$1/R_{ct} = A \exp (E_a/RT)$$

R_{ct} is the charge transfer resistance, Ea is the activation energy, T is the absolute temperature, R is the gas constant, and A is the pre-exponential factor.

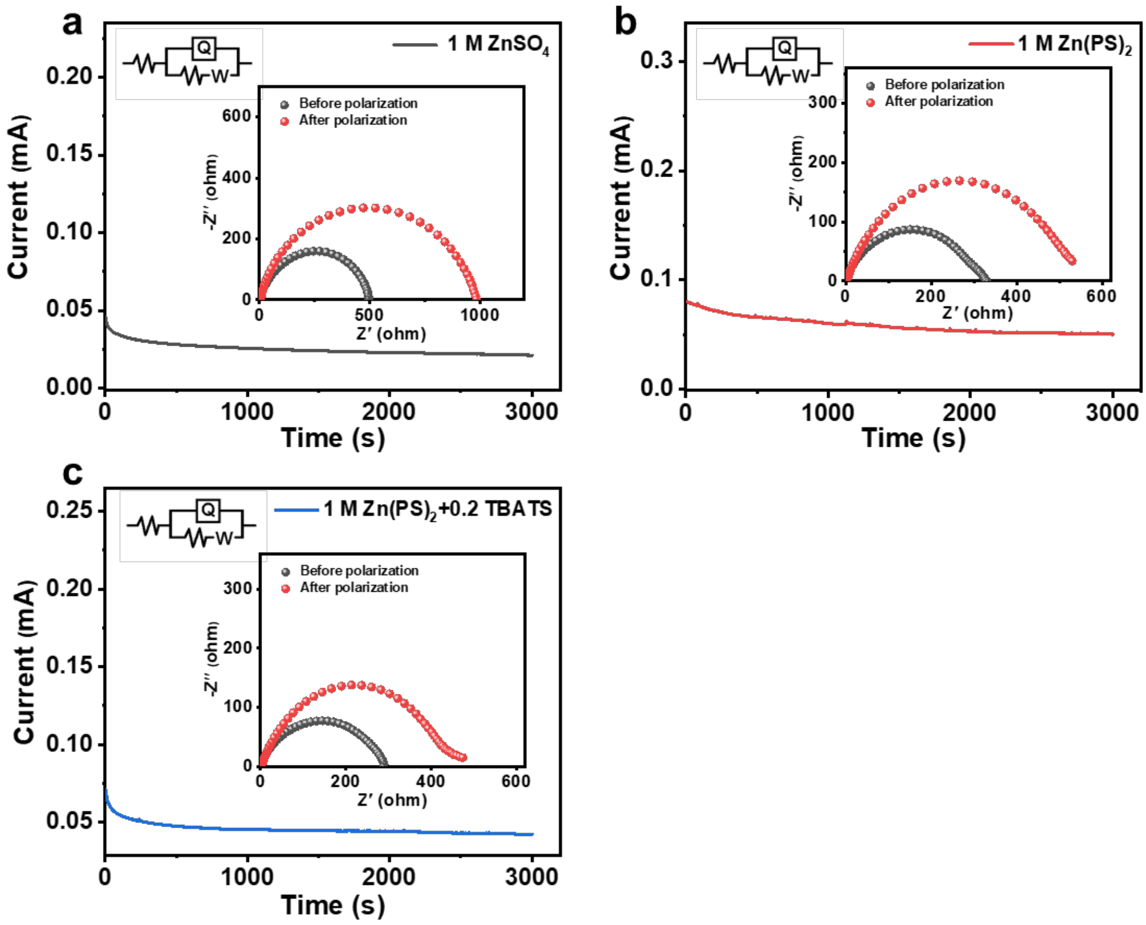

Supplementary Fig. 28 Current-time plots and electrochemical impedance spectroscopy for the measurement of Zn^{2+} transference number in different electrolytes. a) 1 M $ZnSO_4$, b) 1 M $Zn(PS)_2$ and c) 1 M $Zn(PS)_2$+0.2 TBATS electrolytes after polarization at a constant potential (25 mV) for 3000 s. The insets are the equivalent circuit diagram and the impedance spectra before and after the polarization.

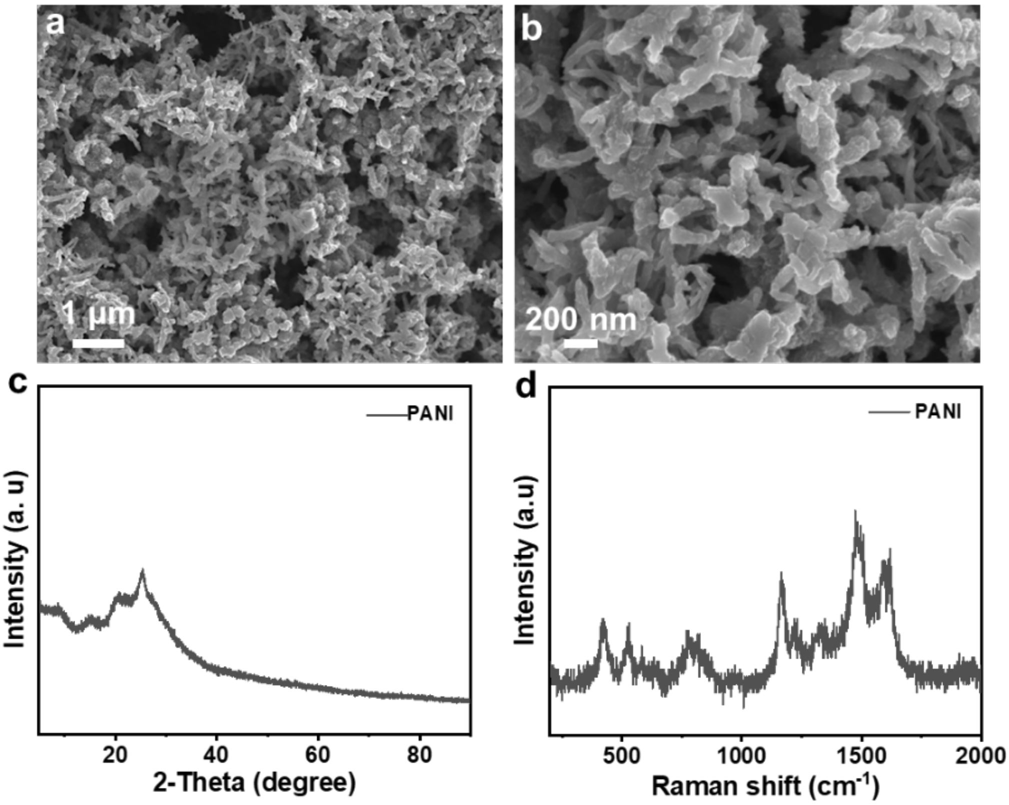

Supplementary Fig. 29 a, b) SEM images, c) XRD pattern and d) Raman spectra of PANI nanofibers.

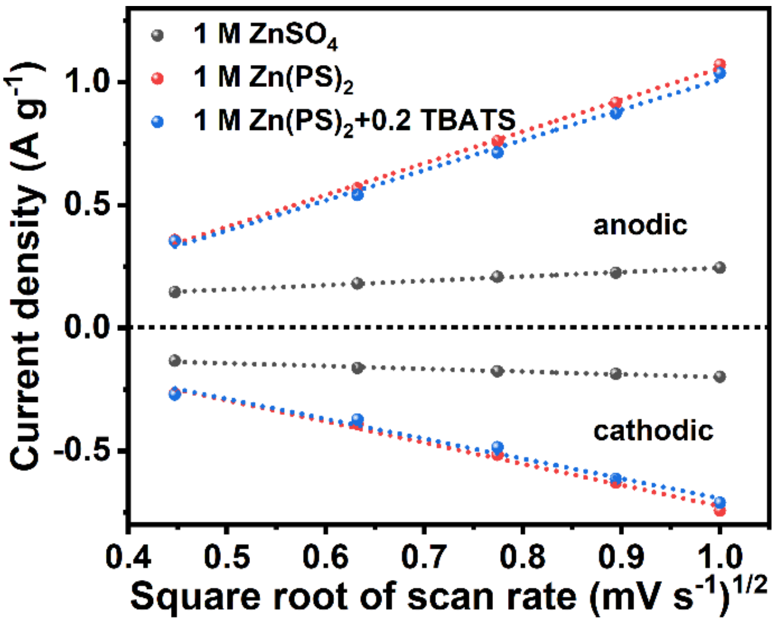

Supplementary Fig. 30 Linear fitting between the peak current and the square root of the scan rates of the CV curves for PANI-Zn batteries.

The diffusion coefficient of Zn^{2+} ions in different electrolytes were calculated according to the Randle-Sevcik equation:

$$I_p=2.69\times10^5 n^{3/2} A D_{Zn}^{1/2} v^{1/2} C_{Zn}$$

where I_p is the peak current, n is the number of electrons, A is the area of the electrode, D_{Zn} is the diffusion coefficient of Zn^{2+} ions, v is the scan rate and C_{Zn} is the concentrate of Zn^{2+} ions. Taking the average value of anodic and cathodic process, the D_{Zn} values are calculated to be 9.53×10^{-9}, 5.22×10^{-7} and 4.69×10^{-7} cm^2 s^{-1} in 1 M $ZnSO_4$, 1 M $Zn(PS)_2$ and 1 M $Zn(PS)_2$+0.2 TBATS, respectively.

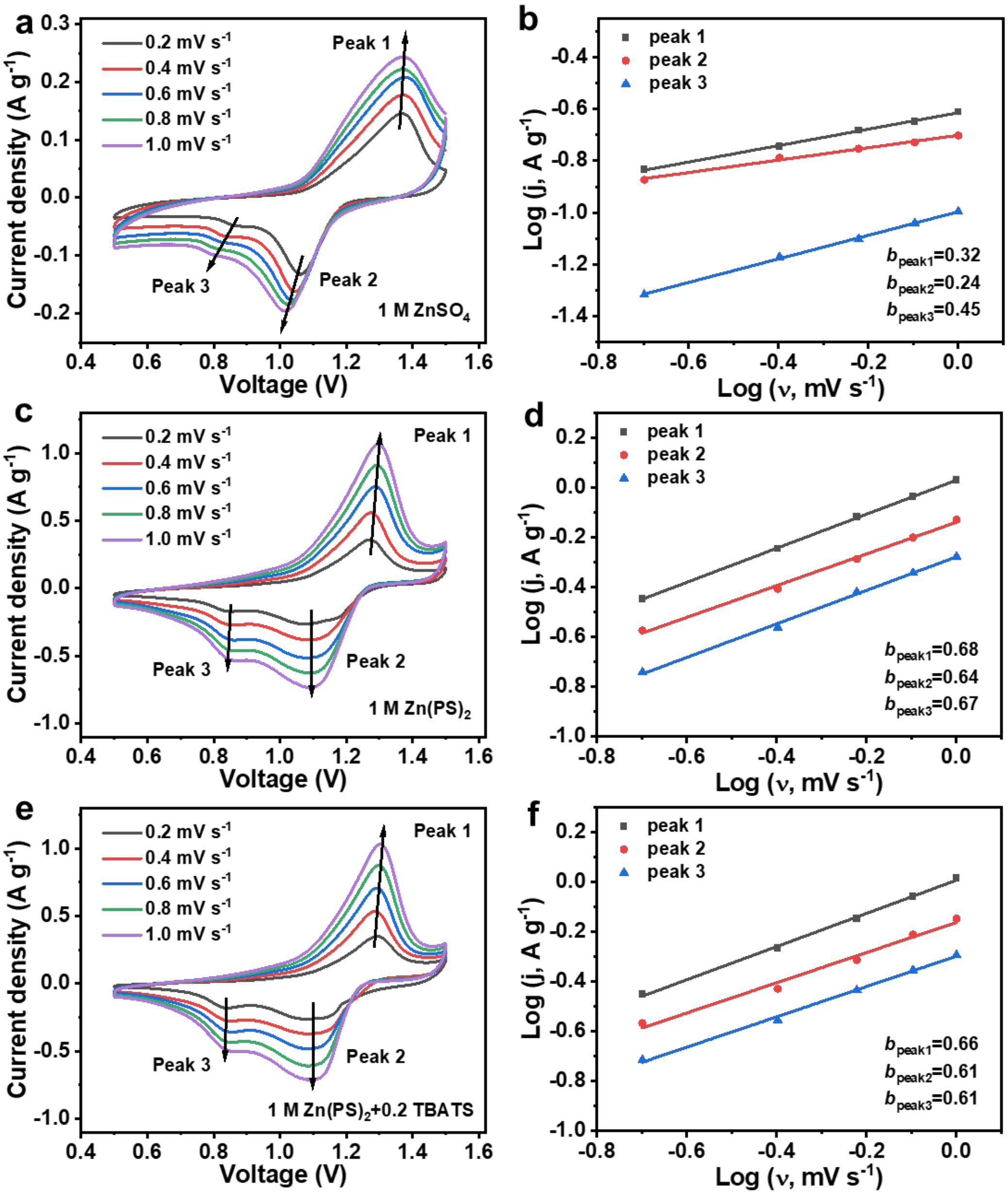

Supplementary Fig. 31 CV curves and corresponding plots of log (peak current) vs log (scan rate) of PANI-Zn batteries with a, b) 1 M $ZnSO_4$ c, d) 1 M $Zn(PS)_2$ and e, f) 1 M $Zn(PS)_2$+0.2 TBATS electrolytes.

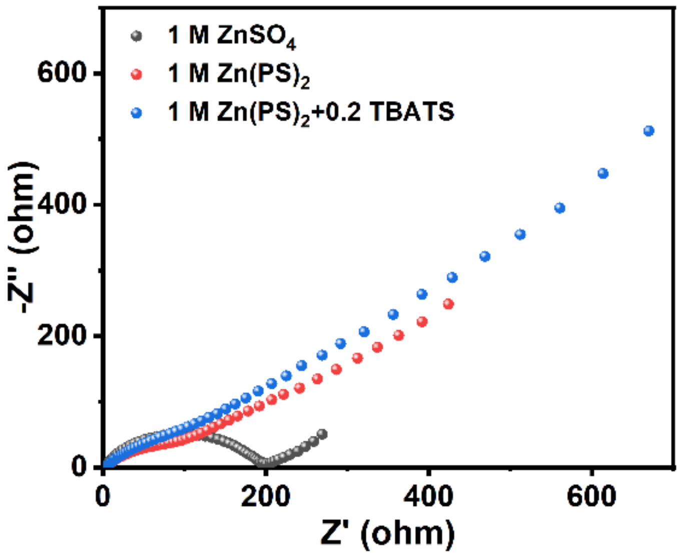

Supplementary Fig. 32 Electrochemical impedance spectra of PANI-Zn batteries at the pristine state.

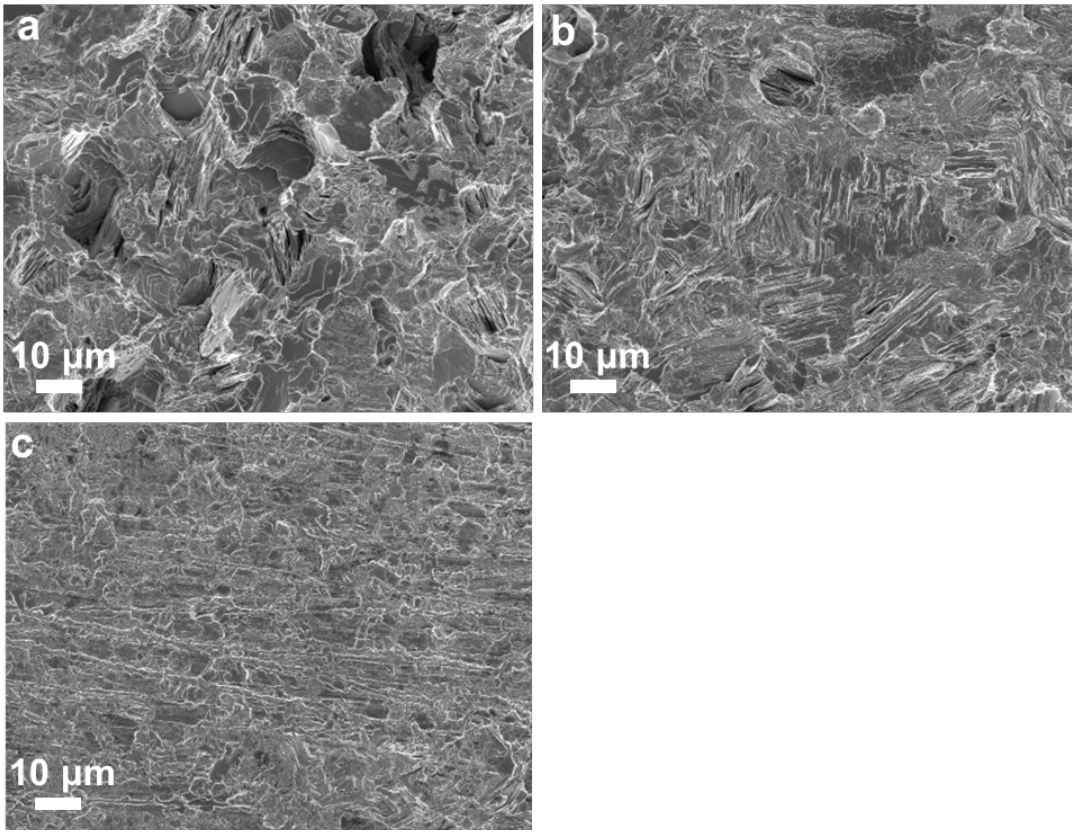

Supplementary Fig. 33 SEM images of Zn anode of PANI-Zn batteries after 1000 cycles with a) 1 M $ZnSO_4$ b) 1 M $Zn(PS)_2$ and c) 1 M $Zn(PS)_2$+0.2 TBATS electrolytes.

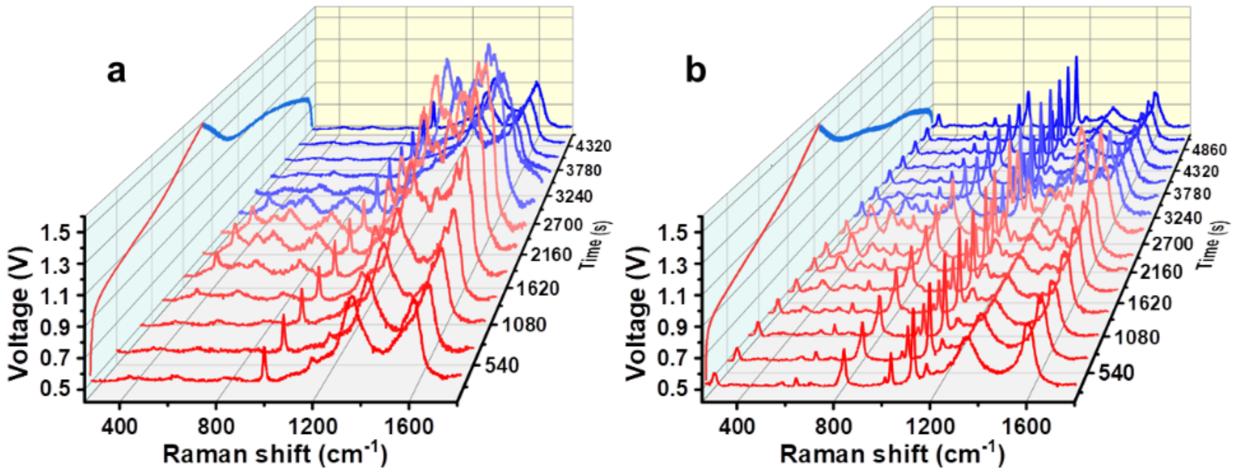

Supplementary Fig. 34 In-situ Raman spectra of PANI cathode in a) 1 M $ZnSO_4$ and b) 1 M $Zn(PS)_2$ electrolytes.

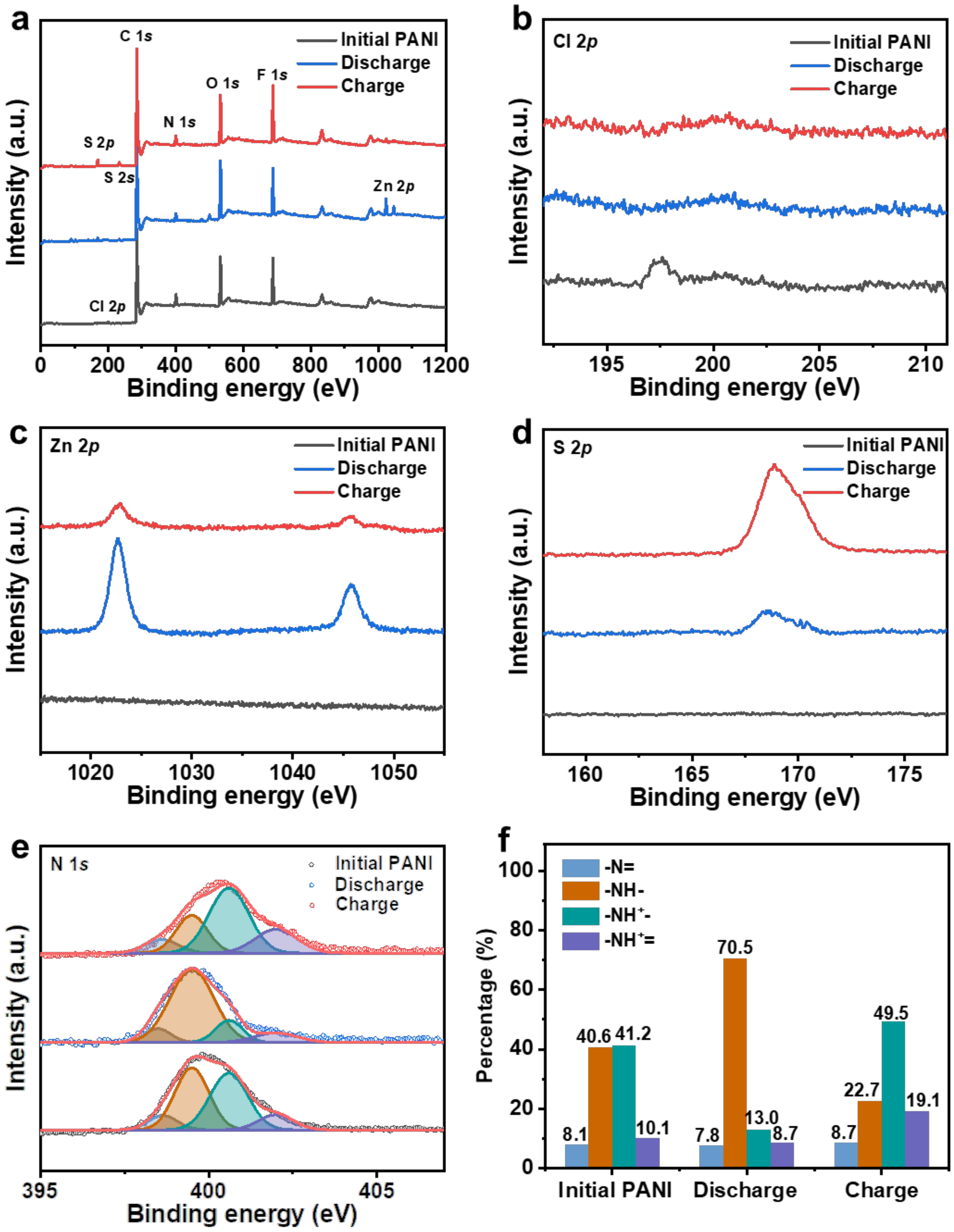

Supplementary Fig. 35 a) The full XPS spectra and core-level spectra of b) Cl 2*p*, c) Zn 2*p*, d) S 2*p*, e) N 1*s* of PANI at various status in 1 M $Zn(PS)_2$+0.2 TBATS electrolyte. f) the corresponding changes of N components.

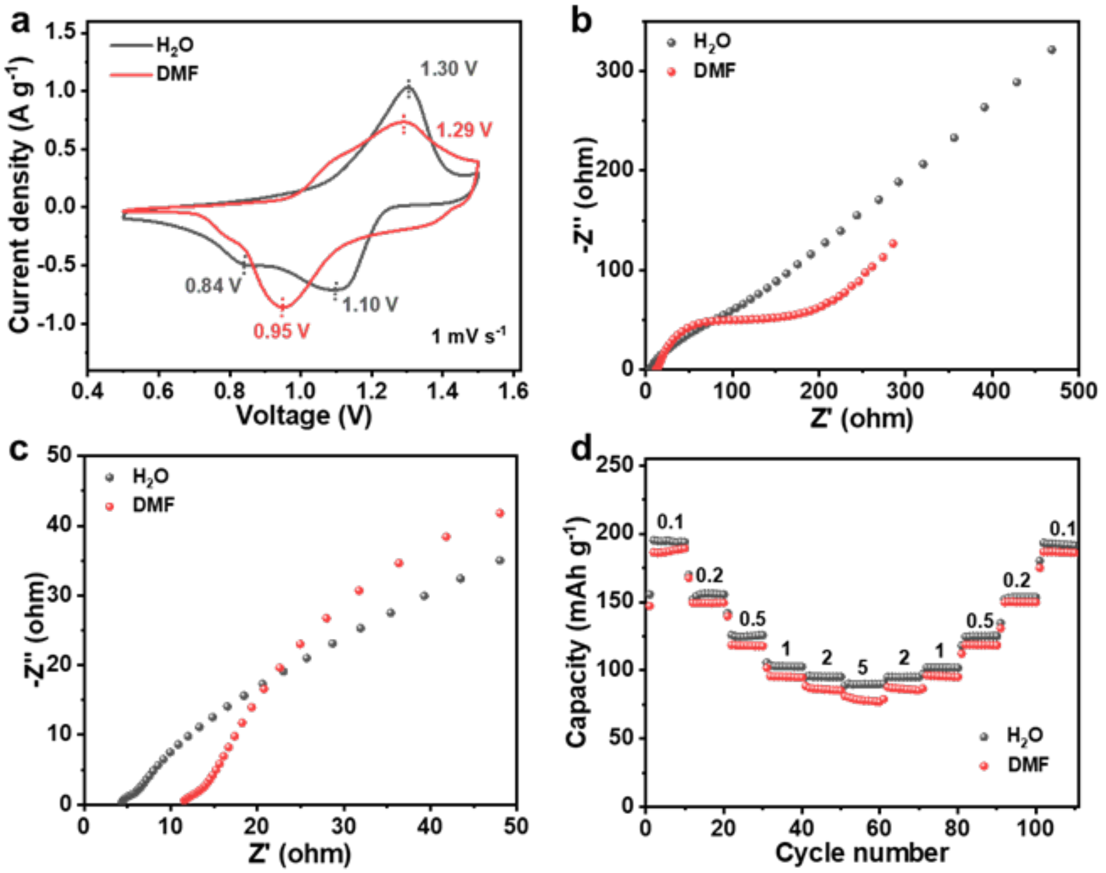

Supplementary Fig. 36 a) Comparison of CV curves of PANI-Zn batteries at 1 mV s^{-1} with 1 M $Zn(PS)_2$+0.2 TBATS electrolyte in H_2O and DMF. b) Nyquist plot of PANI-Zn batteries and c) the enlarged curves in the high-frequency range. d) Rate performance.

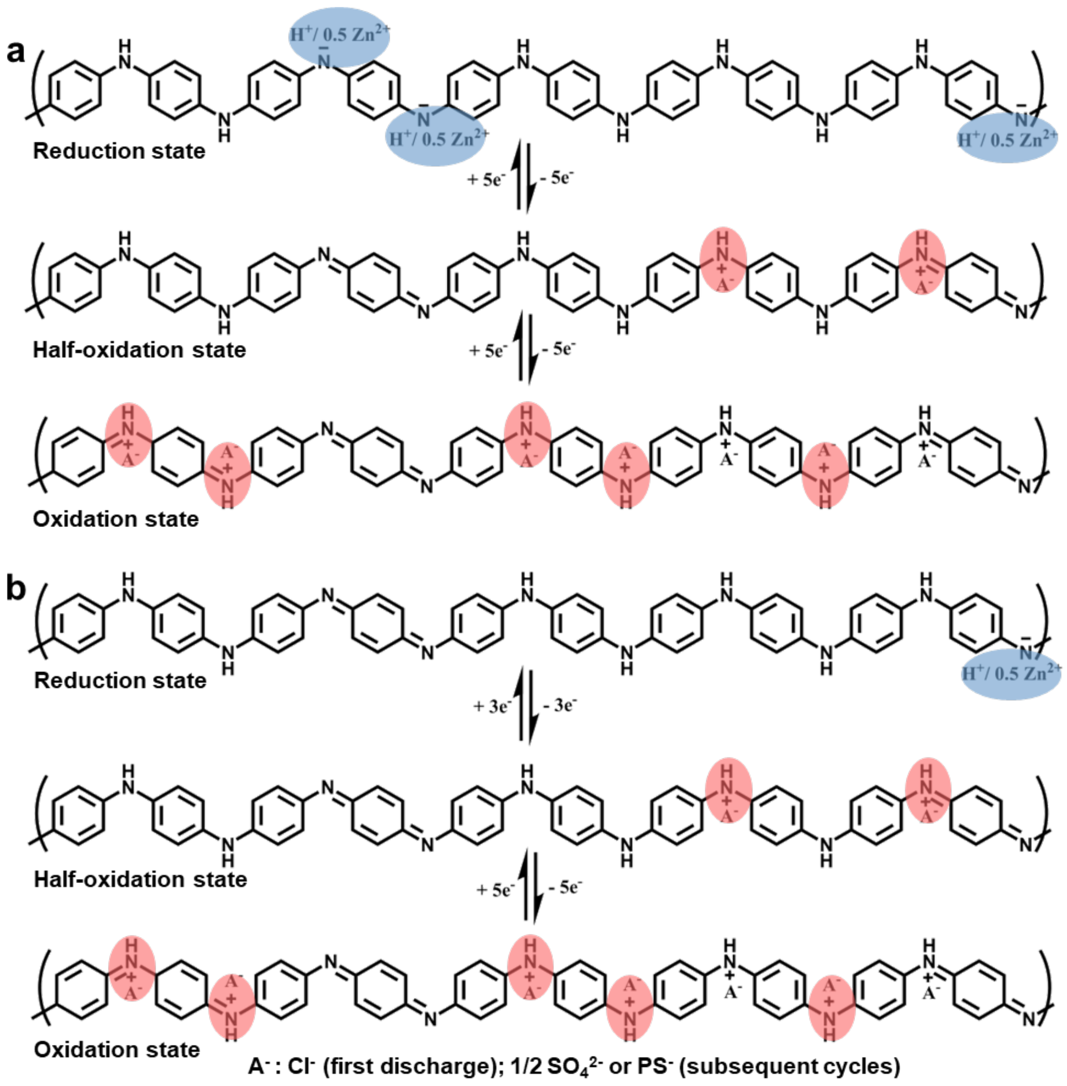

Supplementary Fig. 37 The proposed redox reaction of PANI in a) $Zn(PS)_2$ and b) $ZnSO_4$ electrolytes.

The XPS spectra were performed to examine the component changes during the PANI redox process. As shown in Supplementary Fig. 35a, C, N and Cl elements were detected in the initial PANI, suggesting the successful preparation of PANI. With the absence of Cl^- dopants after the first discharge cycle (Supplementary Fig. 35b), the peaks, corresponding to Zn and S elements respectively are observed (Supplementary Fig. 35c, d). The reuslts suggest that Zn^{2+}, PS^- ions are involved in the energy staroge process. H^+/Zn^{2+} as counterions, are adsorbed onto the reduced polyaniline chain to neutralize the charges on the polyaniline chains. The oxidized groups on the polyaniline chains, such as -NH^+- and -NH^+=, are stabilized by the PS^- anion via the electrostatic interaction. The N 1s can be fitted with four components, i.e., -N= (~398.6 eV), -NH- (~399.5 eV), -NH^+- (~400.6 eV) and -NH^+= (~402 eV). The -NH- component is corresponding to the reduced state and the others are in the oxided status. The amount of oxidized and reduced components are almost equal in accord with the emeraldine state of the polymer at the initial state (Supplementary Fig. 35e, f). Upon the discharge process, the reduced -NH- component increases to 70.5%

with the H^+/Zn^{2+} adsorption. Upon the charge process, polyaniline is oxidized, H^+/Zn^{2+} is desorbed from the polyaniline chains with the desreasing of the reduced -NH- component (22.7%). Meanwhile, the oxidized components, $-NH^+-$ and $-NH^+=$ increase to 49.5 and 19.1%, respectively, accompanied by the adsorption of dopant PS^- to balance charge.

The $Zn(PS)_2$ salt and TBATS additive were dissolved in an aprotic solvent, N, N-dimethylformamide (DMF) to eliminate the H^+ insertion contribution. In the aprotic electrolyte, the CV curves exhibit a pair of redox peaks situated at 1.29 and 0.95 V, corresponding to the redox reactions of PANI (Supplementary Fig. 36a). The low ionic conductivity results in the large potential polarization (Supplementary Fig. 36b, c). In contrast, the smaller polarization in aqueous electrolyte would contribute to the favorable redox process of PANI. The assembled PANI-Zn battery delivers the similar specific capacity at different current density (Supplementary Fig. 36d). Specifically, the specific capacity is 185 mAh g^{-1} at the current density of 0.1 A g^{-1} which is comparable to that in aqueous electrolyte (194 mAh g^{-1}) (Fig. 5b). Obviously, the H^+ insertion is not dominant to the energy storge process of PANI in the present case.

The energy storage mechanism of PANI in $Zn(PS)_2$ electrolyte is proposed according to the in-situ Raman spectra and XPS results (Supplementary Fig. 37a). During the first discharge process, PANI chains with $-NH^+-$ and $-N^+=$ are reduced to -NH-, the dopant (e.g., Cl^-) would be desorbed from the palyanilne chain (Supplementary Fig. 35b). Meanwhile, the -N= in PANI is reduced to $-N^--$ for anchoring the additional H^+/Zn^{2+} ions along with the transformation from quinonoid to benzenoid structure (Fig. 5e). Notably, the easy desolvation in $Zn(PS)_2$ electrolyte leads to the formation of the free Zn^{2+} ions as additional dopants, thus enhancing the specific capacity (Fig. 5b). During the charge process, the adsorbed H^+/Zn^{2+} ions are removed from PANI, the oxidation of -NH- groups into -N=, $-NH^+-$ and $-N^+=$ provides dopant sites for the counterions (e.g., PS^-) adsorbed into PANI chains due to the electrostatic interaction. The gradual presence of typical peaks for quinonoid structure in Raman spectra (Fig. 5e) suggests the good redox reversibility.

In contrast, the calculated desolvation energy of $Zn(H_2O)_5(SO_4)$ in $ZnSO_4$ electrolyte is -719.4 kcal mol^{-1}, which is higher than that of $Zn(H_2O)_5(PS)^+$ in $Zn(PS)_2$ electrolyte (Fig. 3d). Thus, Zn^{2+} ions as counterions are not easily released from $Zn(H_2O)_5(SO_4)$ to balance the charge on PANI chains, which would limits the specific capacity of PANI (Supplementary Fig. 37b).

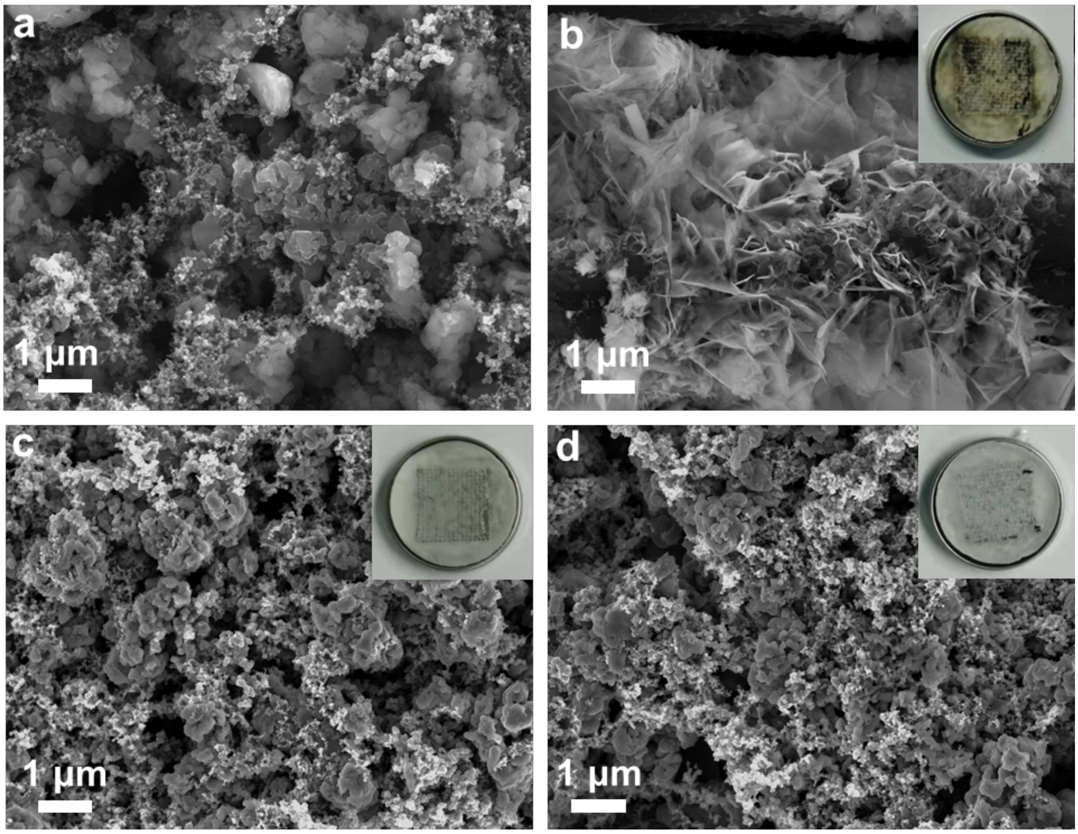

Supplementary Fig. 38 SEM images of PANI cathode at a) initial state and photographs of seperator after 1000 cycles in b) 1 M $ZnSO_4$, c) 1 M $Zn(PS)_2$ and d) 1 M $Zn(PS)_2$+0.2 TBATS electrolytes.

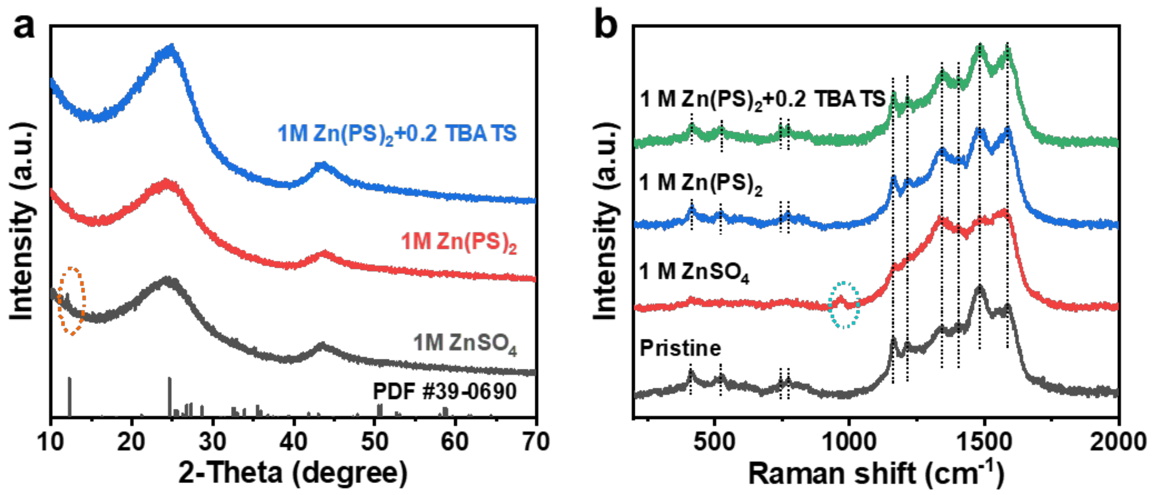

Supplementary Fig. 39 XRD patterns and Raman spectra of PANI cathode after 1000 cycles in different electrolytes.

The newly emerged peak at 12.2° after cycling in 1 M $ZnSO_4$ electrolyte is corresponding to $Zn_4SO_4(OH)_6·H_2O$ formed (Supplementary Fig.39a). The peak appears at 968 cm^{-1} is related to the stretching vibration of SO_4^{2-} (Supplementary Fig.39b). In comparison, the PANI cathode demonstrates the good structure stability after 1000 cycles in $Zn(PS)_2$ electrolyte and no imputy is detected.

2. *Supplementary Tables*

Supplementary Table 1 The voltage hysteresis comparison of Zn-Zn symmetric cell with 1 M $Zn(PS)_2$+0.2 TBATS electrolyte at 1 mA cm^{-2}, 1 mAh cm^{-2} with recently reports.

Electrode/electrolyte	Overpotential 1 (1st cycle)	Overpotential 2	Ref.
1 M $Zn(PS)_2$+0.2 TBATS	22.6 mV	17.7 mV (200th cycle)	This work
Zn@ZnF_2	35.7 mV		[1]
$Zn(H_2PO_4)_2$ additive	40 mV		[2]
TU/$ZnSO_4$		52 mV (27th cycle)	[3]
Cu-Zn@Zn		30 mV	[4]
SPEEK-Zn		50 mV	[5]
$La(NO_3)_3$ additive		<50 mV	[6]
Sn@NHCF-Zn		21 mV	[7]

Supplementary Table 2 Element contents of Zn foil surface immersed in solutions for one week.

Solution	Element	Weight percentage (%)	Atomic percentage (%)
	Zn	69.75	38.60
1 M $ZnSO_4$	O	24.05	54.41
	S	6.20	6.99
	Zn	98.60	94.50
1 M $Zn(PS)_2$	O	1.40	5.50
	S	0.00	0.00
	Zn	99.49	97.97
1 M $Zn(PS)_2$+0.2 TBATS	O	0.51	2.03
	S	0.00	0.00

Supplementary Table 3 Fitting results of Raman spectra of $ZnSO_4$ electrolyte.

Concentration	Wavenumber			
	978 cm^{-1}	982 cm^{-1}	3235 cm^{-1}	3425 cm^{-1}
0.2 M	57%	43%	56%	44%
0.5 M	53%	47%	52%	48%
1 M	45%	55%	44%	56%
saturated	28%	72%	27%	73%

Supplementary Table 4 Fitting results of Raman spectra of $Zn(PS)_2$ electrolyte.

Concentration	Wavenumber	
	3235 cm^{-1}	3425 cm^{-1}
0.2 M	54%	46%
0.5 M	48%	52%
1 M	42%	58%
saturated	26%	74%

Supplementary Table 5 The fitted R_{ct} of Zn symmetric cells in 1 M $ZnSO_4$ electrolyte.

**T** (°C)	$\boldsymbol{R_{ct}}$ (Ω)	**Error** (%)	**ln** ($\boldsymbol{R_{ct}^{-1}}$) (Ω^{-1})
30	246.10	4.42	-5.50
40	128.40	3.64	-4.85
50	68.25	2.01	-4.22
60	31.16	0.30	-3.44
70	14.53	1.37	-2.68

Supplementary Table 6 The fitted R_{ct} of Zn symmetric cells in 1 M $Zn(PS)_2$ electrolyte.

**T** (°C)	$\boldsymbol{R_{ct}}$ (Ω)	**Error** (%)	**ln** ($\boldsymbol{R_{ct}^{-1}}$) (Ω^{-1})
30	181.50	3.14	-5.20
40	108.01	3.25	-4.68
50	60.25	0.49	-4.10
60	31.53	1.40	-3.45
70	17.61	1.32	-2.87

Supplementary Table 7 The fitted R_{ct} of Zn symmetric cells in 1 M $Zn(PS)_2$+0.2 TBATS electrolyte.

**T** (°C)	$\boldsymbol{R_{ct}}$ (Ω)	**Error** (%)	**ln** ($\boldsymbol{R_{ct}^{-1}}$) (Ω^{-1})
30	196.92	2.12	-5.28
40	119.03	2.62	-4.78
50	71.74	2.19	-4.27
60	33.63	0.70	-3.52
70	18.18	1.02	-2.90

Supplementary Table 8 The electrochemical performance comparison of PANI-Zn batteries based on different electrolytes.

Electrolyte	Specific capacity	Capacity retention	Ref.
1 M $Zn(PS)_2$+0.2 TBATS	194 mAh g^{-1} at 0.1 A g^{-1}	82% after 10000 cycles	**This work**
9 M $ZnCl_2$	183 mAh g^{-1} at 0.7 A g^{-1}	83.5% after 1000 cycles	[8]
1 M $ZnSO_4$+4 M EMImCl	154.4 mAh g^{-1} at 1 A g^{-1}	78.8% after 300 cycles	[9]
$ZnCl_2$: EG = 1: 4 (molar ratio)	180 mAh g^{-1} at 0.1 A g^{-1}	78% after 10000 cycles	[10]
1 M $Zn(CF_3SO_3)_2$	191 mAh g^{-1} at 0.05 A g^{-1}	92% after 3000 cycles	[11]
2 M $ZnSO_4$+0.05 M SG	192.3 mAh g^{-1} at 1 A g^{-1}	81.7% after 1400 cycles	[12]
7.5 M $ZnCl_2$	106.2 mAh g^{-1} at 0.02 A g^{-1} (-70°C)	~100% after 2000 cycles (-70°C)	[13]

Supplementary Table 9 Assignment of Raman peaks of the PANI-Zn batteries with different electrolytes.

Wavenumber (cm^{-1})		Assignment
$ZnSO_4$	$Zn(PS)_2$ / $Zn(PS)_2$+0.2 TBATS	
416	416	Ring deformation
526	525	
608	580	
749	749	Imine deformation
778	785	Quinone ring deformation
1170	1168	C-H bending vibrations of quinone ring
1220	1219	C-N stretching vibration of benzene diamine units
1347	1339	C-N^+ stretching vibration of delocalized polaronic structures
1406	1417	C-C stretching vibration of quinone
1482	1492	C=N stretching vibration
1563	1567	C=C stretching vibration of quinone
1598	1589	C-C stretching vibration
1627	1622	

Supplementary references

1. Yang, Y. et al. Synergistic manipulation of Zn^{2+} ion flux and desolvation effect enabled by anodic growth of a 3D ZnF_2 matrix for long-lifespan and dendrite-free Zn metal anodes. *Adv Mater* **33**, e2007388 (2021).
2. Zeng, X. et al. Electrolyte design for in situ construction of highly Zn^{2+}-conductive solid electrolyte interphase to enable high-performance aqueous Zn-ion batteries under practical conditions. *Adv Mater* **33**, e2007416 (2021).
3. Qin, H. et al. Building metal-molecule interface towards stable and reversible Zn metal anodes for aqueous rechargeable zinc batteries. *Adv. Funct. Mater.* **32**, 2206695 (2022).
4. Li, B. et al. Multicomponent copper-zinc alloy layer enabling ultra-stable zinc metal anode of aqueous Zn-ion battery. *Angew. Chem. Int. Ed.* **61**, e202212587 (2022).
5. Fan, H. et al. Tailoring interfacial Zn^{2+} coordination via a robust cation conductive film enables high performance zinc metal battery. *Energy Storage Mater.* **49**, 380-389 (2022).
6. Zhao, R. et al. Lanthanum nitrate as aqueous electrolyte additive for favourable zinc metal electrodeposition. *Nat. Commun.* **13**, 3252 (2022).
7. Yu, H. et al. Confining Sn nanoparticles in interconnected N-doped hollow carbon spheres as hierarchical zincophilic fibers for dendrite-free Zn metal anodes. *Sci. Adv.* **8**, eabm5766 (2022).
8. Yan, H. et al. Protonating imine sites of polyaniline for aqueous zinc batteries. *Chem. Commun.* **58**, 1693-1696 (2022).
9. Zhang, Q. et al. Designing anion-type water-free Zn^{2+} solvation structure for robust Zn metal anode. *Angew. Chem. Int. Ed.* **60**, 23357-23364 (2021).
10. Geng, L. et al. Eutectic electrolyte with unique solvation structure for high-performance zinc-ion batteries. *Angew. Chem. Int. Ed.* **61**, e202206717 (2022).
11. Wan, F. et al. An aqueous rechargeable zinc-organic battery with hybrid mechanism. *Adv. Funct. Mater.* **28**, 1804975 (2018).
12. Hao, J., Yuan, L., Zhu, Y., Jaroniec, M. & Qiao, S. Z. Triple-function electrolyte regulation toward advanced aqueous Zn-ion batteries. *Adv Mater* **34**, e2206963 (2022).
13. Zhang, Q. et al. Modulating electrolyte structure for ultralow temperature aqueous zinc batteries. *Nat. Commun.* **11**, 4463 (2020).
